# Magnetic resonance imaging findings in pediatric neck infections—a comparison with adult patients

**DOI:** 10.1007/s00247-021-05275-6

**Published:** 2022-02-20

**Authors:** Janne Nurminen, Jaakko Heikkinen, Tatu Happonen, Jarno Velhonoja, Heikki Irjala, Tero Soukka, Lauri Ivaska, Kimmo Mattila, Jussi Hirvonen

**Affiliations:** 1 Diagnostic Radiology, University of Turku, Kiinamyllynkatu 4–8, 20520 Turku, Finland; 2 Department of Otorhinolaryngology-Head and Neck Surgery, University of Turku and Turku University Hospital, Turku, Finland; 3 Department of Oral and Maxillofacial Surgery, University of Turku, Turku, Finland; 4 Department of Paediatrics and Adolescent Medicine, University of Turku and Turku University Hospital, Turku, Finland

**Keywords:** Abscess, Adults, Children, Emergency medicine, Infection, Magnetic resonance imaging, Neck

## Abstract

**Background:**

Differences in the functioning of the immune system and the anatomical proportions of the neck between children and adults lead to different manifestations of deep neck infections. Magnetic resonance imaging (MRI) may serve as an alternative to computed tomography (CT) as the primary imaging modality.

**Objective:**

To study characteristic MRI findings and the diagnostic accuracy of MRI in pediatric deep neck infections.

**Materials and methods:**

We retrospectively studied a cohort of pediatric patients who underwent a neck 3-tesla MRI study over a five-year period. Inclusion criteria were: 1) emergency MRI findings indicating an infection, 2) infection as the final clinical diagnosis, 3) diagnostic image quality verified by the radiologist reading the study and 4) age under 18 years. Patient record data, including surgery reports, were compared with the MRI findings.

**Results:**

Data of 45 children were included and analysed. Compared to adults, children had a higher incidence of retropharyngeal infection and lymphadenitis, and a lower incidence of peritonsillar/parapharyngeal infection. MRI showed evidence of an abscess in 34 children. Of these 34 patients, 24 underwent surgery, which confirmed an abscess in 21 but no abscess in three patients. In addition, three patients underwent surgery without MRI evidence of abscess, and an abscess was found in one of these cases. The measures of diagnostic accuracy among the children were sensitivity 0.96, specificity 0.77, positive predictive value 0.89, negative predictive value 0.91 and accuracy 0.89. Compared with adults, children had lower C-reactive protein, but a similar proportion of them had an abscess, and abscess size and rate of surgery were similar.

**Conclusion:**

Despite the differences in the infection foci, emergency MRI in children had equal diagnostic accuracy to that in adults.

**Supplementary Information:**

The online version contains supplementary material available at 10.1007/s00247-021-05275-6.

## Introduction

Deep neck infections are defined as infections that spread along the fascial planes and spaces of the head and neck [1]. Differences in the anatomical proportions of the neck, as well as the proportional distribution and function of the neck lymph nodes between children and adults [2], may lead to dissimilar manifestations of the disease process [3]. In adults, lymph nodes located in the retropharyngeal space, for example, tend to undergo atrophy and are thus not involved in infectious diseases to the same extent. Further, the clinical presentation and microbiology of deep neck space infections seem to vary between children and adults [4].

Deep neck infections and associated abscesses pose a risk of serious complications in children as well as adults, including airway compromise, vascular complications, septicemia and mediastinitis. Deep neck abscesses are often treated with intravenous antimicrobial drugs and surgical incision and drainage. In clinical practice, determining whether the symptoms and findings reflect tissue cellulitis or phlegmon rather than true abscess formation can be challenging. Imaging can aid the diagnostic process by differentiating findings between cellulitis and abscess and may thus suggest conservative treatment as the primary choice if no abscess is detected. It has been estimated that 10–15% of deep neck infections in pediatric patients could be successfully treated with intravenous antibiotics alone [5–7]. Precise determination of abscess location and extension, and the exclusion of possible life-threatening complications, also helps in planning the correct treatment.

Computed tomography (CT) is considered the primary imaging method for neck infections, and previous literature on the medical imaging of deep neck infections in children has therefore focused on CT [8–10]. CT diagnosis of an abscess is usually based on the appearance of a bulging fluid collection with a low-density core and an enhancing rim. However, the positive predictive value (PPV) for detecting abscesses using CT is only about 0.80 in larger studies with surgical reference standards [8, 9, 11–15]. The low PPV of CT imaging may predispose patients to unnecessary surgeries, whereas false negatives can delay appropriate surgical treatment. Magnetic resonance imaging (MRI) is not based on ionizing radiation and could therefore be considered advantageous, especially among children. Recently, emergency neck MRI proved to be feasible and to have high accuracy in detecting deep neck abscesses in a mostly adult sample [16], exceeding the previously found accuracy of CT in adults [17]. Thus far, it is unknown whether MRI findings and diagnostic accuracy are similar among adults and children.

The aim of this retrospective cohort study was to characterize the MRI findings in children with neck infections and to compare them with those previously found in adults [16]. We further compared the MRI findings with surgical reports as a reference standard, to calculate the diagnostic accuracy of MRI in detecting neck abscesses in children.

## Materials and methods

The overall study setting and MRI protocol have been previously published [16]. In the current study, we conducted a secondary study of this data, focusing on pediatric patients who had undergone an MRI of the neck soft tissues between 1 April 2013 and 31 December 2018. We obtained permission from the hospital district board but did not need to obtain written patient consent due to the retrospective nature of the study. We sought no institutional review board (IRB) review (approval or waiver) because the national legislature does not require this for retrospective studies of existing data. The inclusion criteria were: 1) emergency MRI with evidence of infection, 2) infection as the final clinical diagnosis, 3) diagnostic image quality verified by the radiologist reading the study and 4) age < 18 years. The pediatric data were compared to adult data (18 years of age or older) from the same period (i.e. using inclusion criteria 1–3). Demographic, clinical and laboratory variables extracted from medical records included: age (years), sex (male/female), body mass index (BMI kg/m^2^), duration of symptoms before imaging (days), plasma C-reactive protein (CRP, mg/L) level, white blood cell count (10^9^/L), body temperature at admission (°C), surgery (yes/no), treatment details related to sedation or general anesthesia during MRI scanning (medications, intubation, etc.), treatment at an intensive care unit (ICU, yes/no) and length of hospital stay (days).

The MRI protocol was either a combination of axial T2-weighted, fat-suppressed coronal T2-weighted, sagittal T1-weighted, axial diffusion-weighted imaging, fat-saturated axial T1-weighted post-contrast and coronal T1-weighted post-contrast (13% of patients) or a combination of axial T1-weighted, axial T2-weighted Dixon, coronal T2-weighted Dixon, axial diffusion-weighted imaging, and axial, coronal and sagittal T1-weighted Dixon post-contrast (87% of patients). There were no differences in terms of which protocol was used between children and adults (*P* = 0.577). Details of MRI sequences are given in the Online Supplementary Material 1. We routinely administered a gadolinium-based contrast agent (Dotarem; Guerbet, Villepinte, France). To assess the diagnostic accuracy of the MRI findings, we used the following clinical reference standards: For infection, we used the final clinical diagnosis on the medical record; for abscesses, we used the presence or absence of purulence or abscess cavity at surgery if surgery was carried out within 48 h after the MRI. Methods of surgical proof included open surgery, drainage or puncture of pus. Because a patient without an abscess is much less likely to undergo surgery than a patient with an abscess, we considered patients with no abscesses who recovered uneventfully following conservative treatment (including intravenous antibiotics) to represent true negatives.

We defined imaging evidence of infection as any significant high signal on fat-suppressed T2-weighted images consistent with edema, as well as a high signal on fat-suppressed post-contrast T1-weighted Dixon images consistent with abnormal tissue enhancement [16]. More specifically, we defined phlegmon as abnormally enhancing and/or edematous tissue; suppurative lymphadenitis as a lymph node with a non-enhancing center with restricted diffusion; and abscess as a non-enhancing, T2-hyperintense collection with a low apparent diffusion coefficient (restricted diffusion) surrounded by abnormal tissue enhancement [16]. Infected lymph nodes (lymphadenitis) were defined as enlarged nodes with surrounding soft-tissue edema. Suppurative lymphadenitis was considered an intranodal abscess, and infected cystic masses were considered abscesses if they contained purulent fluid, indicated by restricted diffusion. A previous publication examined diagnostic accuracy in a cohort including both children and adults [16]. Of all patients presented in the current study, 310 patients (84%) were included in that paper, 33 children (73%) and 277 adults (85%).

The results are expressed as percentages, means and standard deviations. We compared continuous variables using Student’s *t*-tests, and ordinal variables using chi-square (*Χ*^2^) tests. To locate infection, a significant omnibus chi-square test was followed by regional post hoc comparisons, using adjusted standardized residuals. To assess the diagnostic accuracy of abscess detection, we formulated 2 × 2 tables (MRI vs. surgery) and calculated diagnostic accuracy as previously described [16]. Accuracy was defined as the proportion of true MRI diagnoses (positive or negative) for abscesses among all MRI assessments. The data were analyzed using IBM SPSS Statistics for Mac (version 26, IBM Corporation, Armonk, NY). P-values < 0.05 were considered statistically significant.

## Results

During the study period, 45 pediatric patients underwent emergency MRI for suspected deep neck infection (mean age: 9 years). During the same five-year period, only one pediatric patient underwent CT – a 16-year-old boy with claustrophobia who favored a CT scan over MRI. None of the pediatric patients had to be ruled out from the study because of exclusion criteria. A total of 45 pediatric patients (Table 1) were compared with 326 adults.

**Table 1:** Characteristics of pediatric and adult neck infection patients

Patient characteristics			
	Pediatric	Adult	*P*-value
Sample size (*n*)	45	326	
Age (mean)	9.1	46	< 0.001
Female *n* (%)/male *n* (%)	14 (31)/31 (69)	131 (40)/ 195 (60	0.24
Duration of symptoms at admission (mean, days)	4.8	5.1	0.72
Fever (body temp. 38 ºC or higher) before imaging [*n* (%)]	20 (44)	167 (51)	0.39
White blood cell count (mean, × 10^9^/L)	14.2	14.7	0.81
C-reactive protein value at admission (mean, mg/L)	78.8	127	< 0.001
Hospital stay (mean, days)	3.9	4.5	0.47
Required intensive care [*n* (%)]	10 (22)	40 (12)	0.07
Duration of intravenous antimicrobial medication (mean, days)	4.1	4.4	0.55
Primary infection location [*n* (%)]	45 (100)	326 (100)	< 0.001
*retropharyngeal/mediastinal*	11 (24)	11 (3)	< 0.001
*infected lymph node/cyst*	11 (24)	24 (7)	< 0.001
*peritonsillar/parapharyngeal*	10 (22)	143 (44)	0.005
*sublingual/submandibular*	7 (16)	75 (23)	0.23
*superficial*	3 (7)	10 (3)	0.23
*parotid*	2 (4)	10 (3)	0.62
*masticator/buccal*	1 (2)	36 (11)	0.07
Maximum abscess diameter (mean, mm)	31	35	0.32

**Fig. 1:**
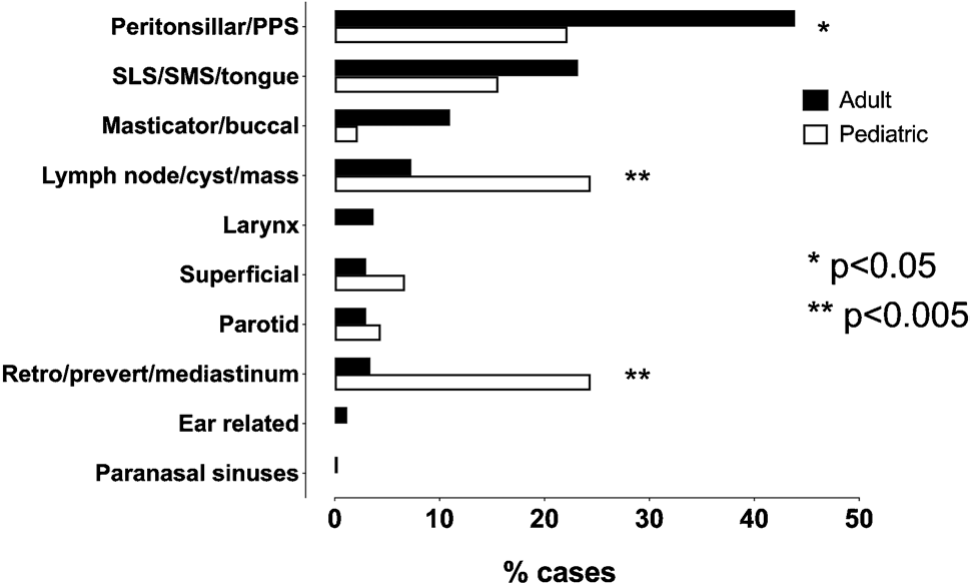
Localization of infections in pediatric and adult patients

Overall, the anatomical distribution of the location of infection was significantly different among the children compared to adults (*X*^2^ = 54, *P* < 0.001) (Fig. 1). Post hoc tests indicated that retropharyngeal space infections were more common among the children (11/45, 24%) than the adults (11/326, 3%), as were infected lymph nodes (24% vs. 7.3%). In contrast, peritonsillar/parapharyngeal infections were more common in adults (143/326, 44%) than in children (10/45, 22%) (Fig. 1). Infections of dental origin were also more frequent among adults (31%) than children (11%). There were further, more subtle differences in the distribution of infection location in the pediatric study group (ages 0–17), especially concerning retropharyngeal space infections and lymphadenitis. Of the 20 patients ages 7 or younger, only one had a main location other than the retropharyngeal space or lymphadenitis. Further, no retropharyngeal space infections were observed among the patients ages 8 to 17 (*n* = 25), and only two had lymphadenitis in this age group. This pattern of results suggests that retropharyngeal infections and lymphadenitis tend to occur in younger children.

**Fig. 2:**
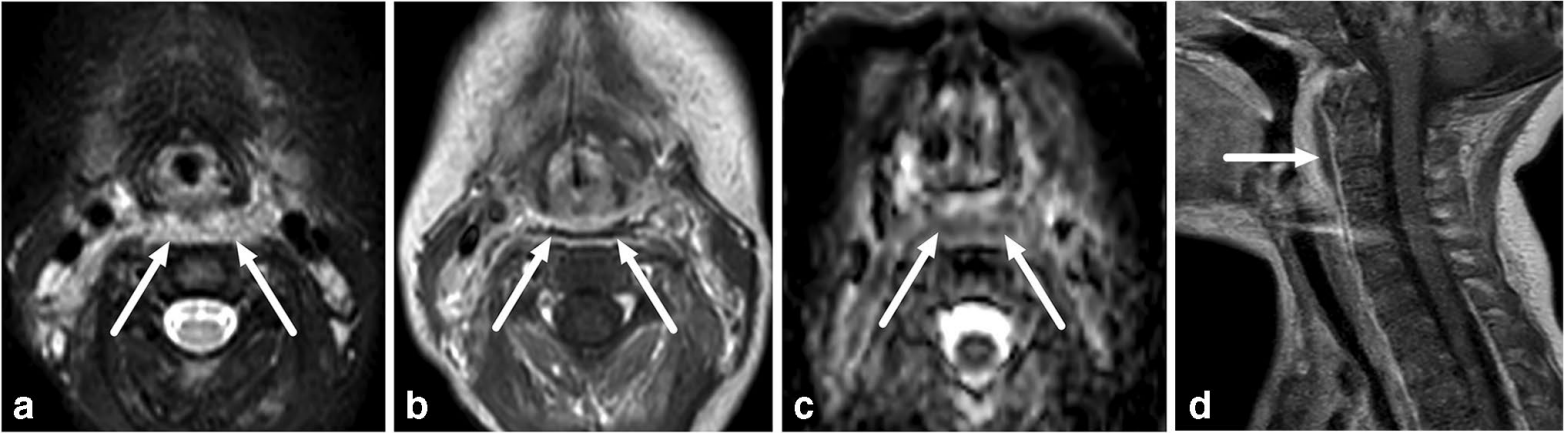
True retropharyngeal abscesses in a 10-month-old boy with a sore throat, who had a thin abscess (*arrows*) that continued caudally into the mediastinum. **a** Axial T2-weighted with fat saturation. **b** Axial T1-weighted post-contrast. **c** Axial apparent diffusion coefficient. **d** Sagittal T1-weighted post-contrast

**Fig. 3:**
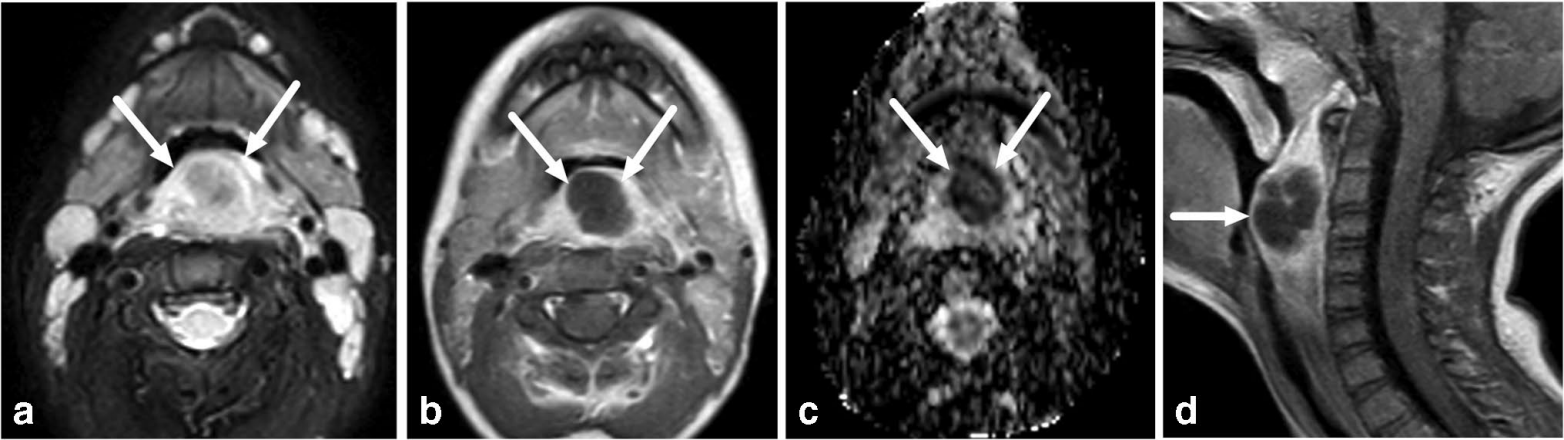
True retropharyngeal abscesses in a 3-year-old boy with throat pain and swelling, who had a bulging abscess (*arrows*) in the median retropharyngeal space. **a** Axial T2-weighted with fat saturation. **b** Axial T1-weighted post-contrast. **c** Axial apparent diffusion coefficient. **d** Sagittal T1-weighted post-contrast

**Fig. 4:**
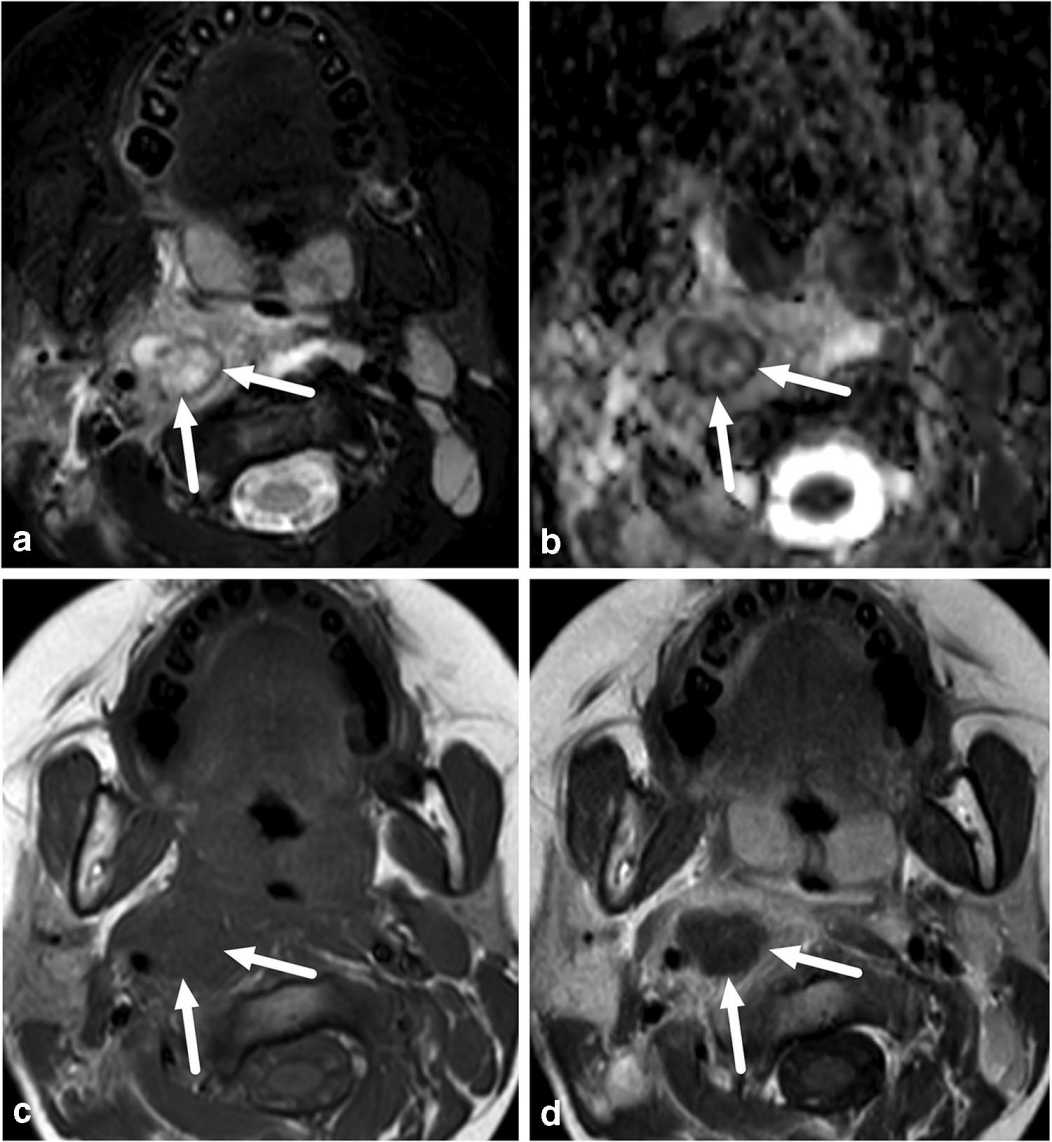
Retropharyngeal suppurative lymphadenitis in a 7-year-old girl with a sore throat and streptococcal tonsilitis. **a–d** Axial images show a focal mass in the right lateral retropharyngeal space (*arrows*) with an intermediate T2-signal (**a**), low apparent diffusion coefficient consistent with restricted diffusion (**b**) and no central contrast enhancement (**c** T1-weighted, before contrast; **d** T1-weighted after contrast), consistent with suppurative lymphadenitis. Purulence was found during surgery

When the retropharyngeal space infections (*n* = 11) were examined in detail, we found that the infection origin was likely a true primary abscess in the retropharyngeal space in only two cases (Fig. 2 and 3). In the other detected retropharyngeal infections, the origin was likely suppurative lymphadenitis (*n* = 9), originating from lateral retropharyngeal nodes (Fig. 4).

**Fig. 5:**
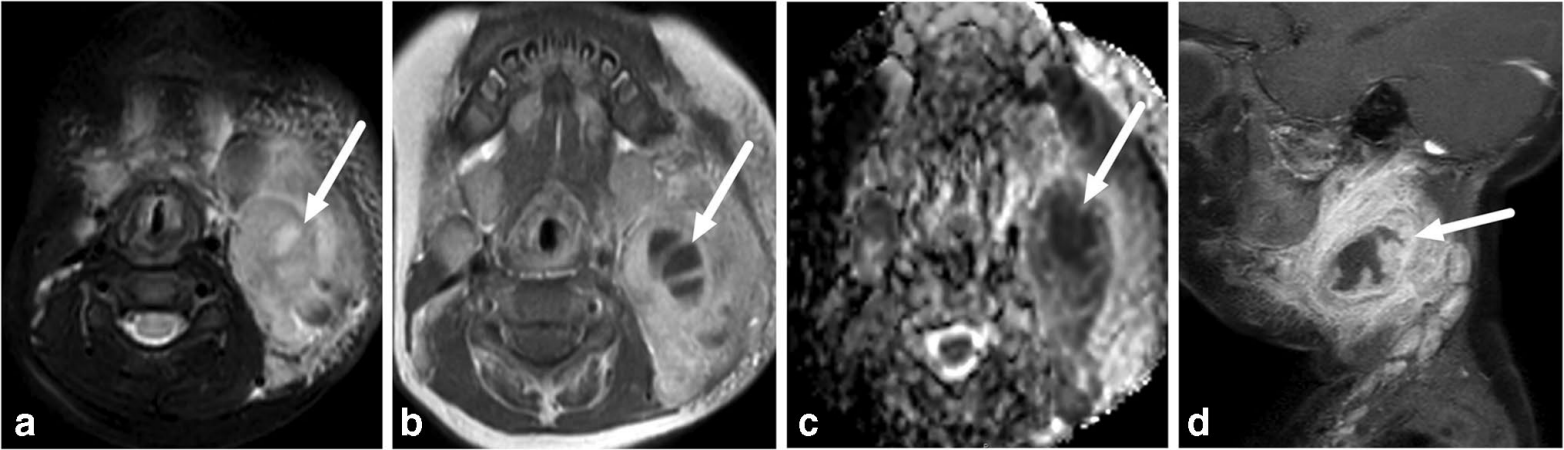
Lateral superficial *Staphylococcus aureus* suppurative lymphadenitis in an 11-month-old boy. Images show a large enhancing lateral neck mass (**a–d** *arrows*) and surrounding tissue edema. Contrast-enhanced images reveal a non-enhancing fluid collection (**b, d** *arrows*) amidst the mass with low apparent diffusion coefficients (**c**), consistent with suppurative lymphadenitis. Purulence was found during surgery and *Staphylococcus aureus* in pus culture. **a** Axial T2-weighted with fat saturation. **b** Axial T1-weighted post-contrast. **c** Axial apparent diffusion coefficient. **d** Sagittal T1-weighted post-contrast

In addition to the cases of lymphadenitis in the retropharyngeal space, we observed 10 other lymphadenitis cases. These represented lateral or superficial infections of the lymph nodes (Fig. 5). Other cases of superficial infections consisted of intramuscular abscess (*n* = 1), cellulitis (*n* = 1) and subcutaneous edema of infectious origin (*n* = 1). Among the children, the only infected mass-like lesion other than a lymph node was the infection of a second branchial cleft cyst (*n* = 1).

**Fig. 6:**
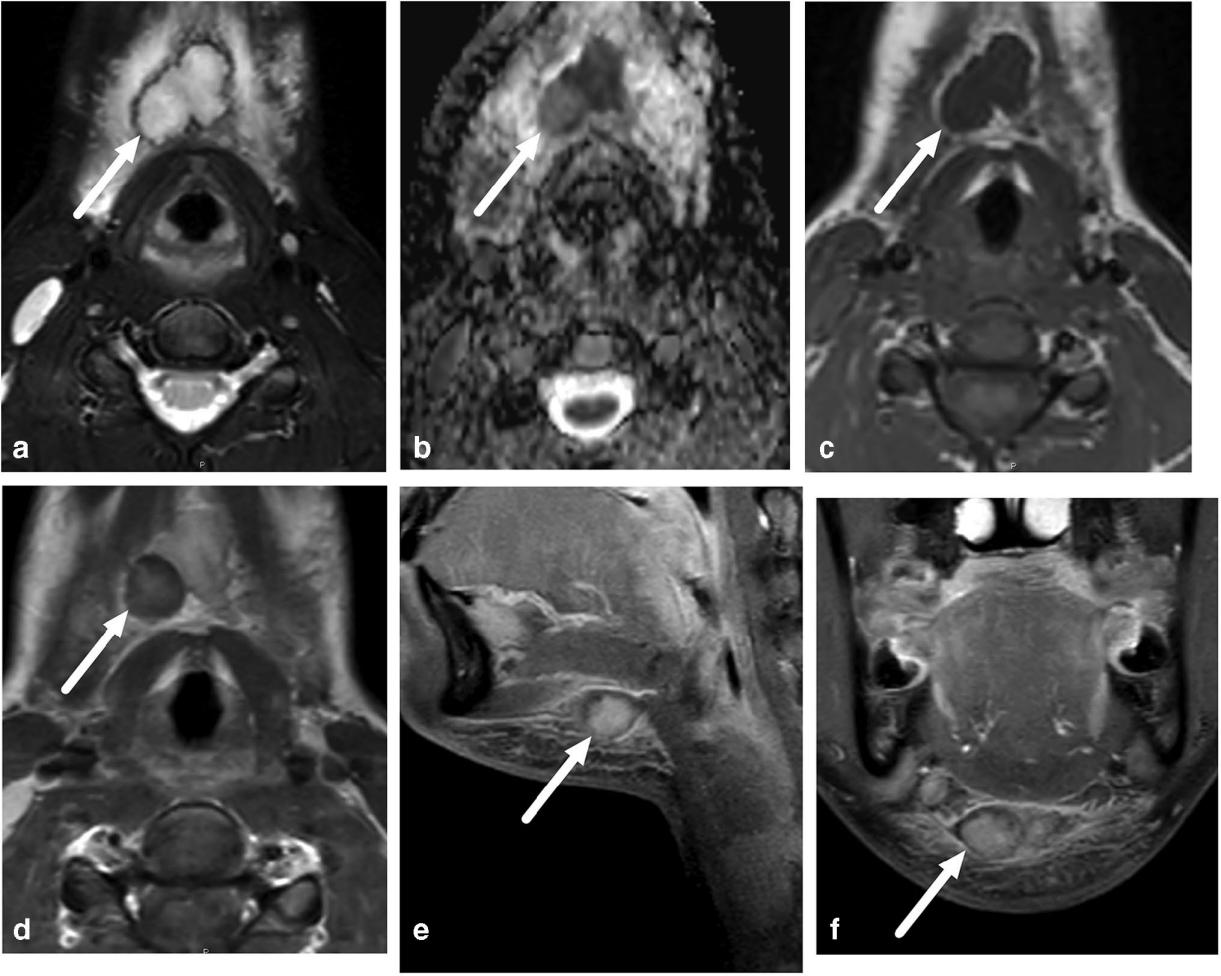
False-positive abscess in a 15-year-old girl with neck swelling. **a** Axial fat-saturated T2-weighted magnetic resonance imaging (MRI) slices (top left) demonstrates lymphadenitis (*arrow*) and surrounding tissue edema. **b** Axial apparent diffusion coefficient image shows restricted diffusion in the lesion (*arrow*). **c, d** Axial T1-weighted images before (**c**) and after (**d**) contrast shows faint partial contrast enhancement compared with pre-contrast image (*arrows*). **e, f** Sagittal (**e**) and coronal (**f**) fat-saturated post-contrast T1-weighted images confirm faint enhancement (*arrows*). This finding was interpreted as suppurative lymphadenitis (intranodal abscess). Surgery found necrotic lymphadenitis, but no purulence. This case highlights the difficulties involved in distinguishing necrosis from early abscess

**Fig. 7:**
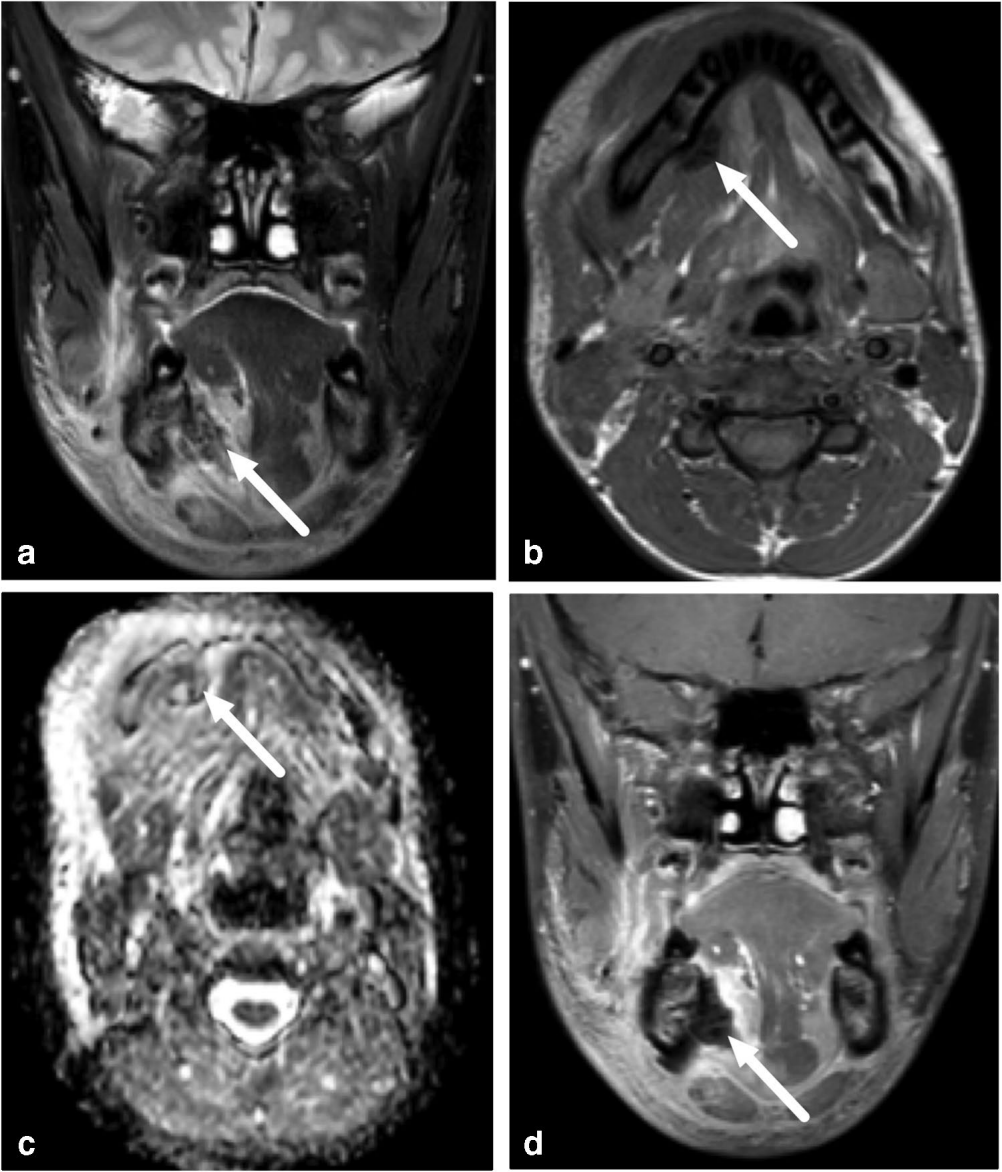
False-negative abscess in a 13-year-old boy with an odontogenic infection. Imaging was done after removal of the infected d46 tooth and incision of an abscess. Coronal fat-saturated T2-weighted (**a**) and axial T1-weighted (**b**) magnetic resonance imaging (MRI) images demonstrate extensive soft-tissue edema, and a relatively T2 and T1 hypointense subperiosteal collection (*arrows*). Axial apparent diffusion coefficient images (**c**) and post-contrast coronal fat-saturated T1-weighted images (**d**) show no restricted diffusion or contrast enhancement (*arrows*). This heterogeneous subperiosteal collection was considered to contain postoperative gas and/or blood with no clear evidence of a residual abscess. Yet, purulence was found at surgery

Of the 45 pediatric patients, 34 had an abscess. The proportion of children with MRI evidence of an abscess (34/45, 76%) did not statistically significantly differ from the proportion of adults (240/326, 74%) (*X*^2^ = 0.08, *P* = 0.78). In total, 27 pediatric patients with evidence of an abscess underwent surgery, which confirmed abscesses in 24 patients. In the case of three patients, however, no abscess was found at surgery, giving a PPV of 0.89 (Table 2). Figure 6 presents an example of such a case. In addition, three patients with no abscess detected on imaging underwent surgery on clinical grounds. Of these, one had an abscess and two did not. Among the children, sensitivity, specificity and accuracy were 0.96, 0.77 and 0.89, respectively. Among the adults, the corresponding values were 0.98, 0.88 and 0.95 (Table 2). The proportion of children (85%) and adults (79%) with MRI evidence of abscesses who underwent surgery did not significantly differ (*X*^2^ = 0.57, *P* = 0.45). Although the proportion of false-positive abscesses was higher among children (11%) than adults (5%), this difference was not statistically significant (*X*^2^ = 1.3, *P* = 0.25). In children, we found 3 false-positive cases: 1) a 7-year-old boy with a parapharyngeal abscess on MRI, but no purulence at surgery (tonsillectomy and explorative neck incision); 2) a 15-year-old girl with submandibular suppurative lymphadenitis on MRI, but only lymphadenitis at surgery (Fig. 6), and 3) a 4-year-old girl with a parapharyngeal abscess on MRI, but no purulence at repeated surgery (a second operation was conducted when repeat MRI showed a persistent abscess after the first operation). In addition, we found one false-negative case (Fig. 7). Regarding abscess size, the average maximal diameter for surgically confirmed abscesses was 31 mm (range: 15–115 mm); respective numbers for conservative treated abscesses were 34 mm (11–120 mm) and 18 mm (17–20 mm) for false-positive abscesses.

**Table 2:** Diagnostic accuracy of magnetic resonance imaging (MRI) in neck abscesses in pediatric and adult patients using surgery report as reference standard

	Pediatric (*n* = 38)	Adult (*n* = 290)
True positive	24	191
False negative	1	4
False positive	3	11
True negative	10	84
Sensitivity	0.96	0.98
Specificity	0.77	0.88
Positive predictive value	0.89	0.95
Negative predictive value	0.91	0.95
False-positive rate	0.23	0.12
False-negative rate	0.04	0.02
Accuracy	0.89	0.95

The pediatric patients had statistically significantly lower initial CRP levels in their plasma than the adults. However, there were no differences in terms of white blood cell count, the duration of symptoms, maximal abscess diameter, length of intravenous antibiotic treatment or length of hospital stay between children and adults (Table 1).

In total, 20 (44%) patients had fever, defined as a body temperature of 38 ºC or higher before the MRI. The majority of the patients (*n* = 41, 91%) received antipyretics before the MRI, and most (24 out of 41) had a combination of both acetaminophen and nonsteroidal anti-inflammatory drugs. Three patients received additional antipyretics during the MRI scan.

In our cohort, 16 (36%) patients were sedated (with spontaneous breathing) and only 3 (7%) required general anesthesia with intubation. All three patients under general anesthesia were transferred to either the operating room or the ICU immediately after the MRI. Fifteen patients were sedated with thiopental and one patient with dexmedetomidine. According to patient records, no immediate complications or adverse events related to sedation or general anesthesia occurred. There were no failed attempts at non-sedated MRI scans.

Six of the pediatric patients (13%) had an underlying health condition: chronic otitis media, developmental chylothorax, rheumatoid arthritis, pulmonary valve stenosis, prematurity, and attention-deficit/hyperactivity disorder.

## Discussion

A previous study has shown that MRI is a feasible imaging method for diagnosing deep neck infections in an emergency care setting, and that it has high diagnostic accuracy for infections and abscesses [16]. Following the current retrospective sub-analysis of the same data, we conclude that MRI is a suitable alternative to CT for diagnosing pediatric deep neck infections.

There are no previously published systematic evaluations of MRI findings of pediatric deep neck infections. Most literature, diagnostic algorithms and recommendations are related to the use of CT as the primary modality among children when diagnostic imaging is required [18].

The most important clinical question for patients with deep neck infection is if there is an abscess and if it is drainable. CT has only a limited ability to differentiate between cellulitis or phlegmon and abscess. In studies on pediatric patients and surgical correlation, this results in a high number of false-negative and false-positive findings, and an overall accuracy of about 0.6–0.8 [8, 9]. A head-to-head comparison has shown MRI to be superior to CT in lesion conspicuity and determination of the number of affected spaces [17]. MRI has previously shown excellent diagnostic accuracy in verifying abscesses [16], and now these findings seem to also apply to children, despite children having clinically dissimilar locations of infection.

Some of the false-positive abscesses in children highlight the need to understand how the proportional anatomical, as well as functional differences between children and adults result in different pathologies. Differentiation between actual suppurative lymphadenitis (intranodal abscess formation) and lymphadenitis may be difficult even with MRI (Fig. 6). Suppurative lymphadenitis is much less common among adults than children. The proportional distribution of lymph nodes is different in children and in the retropharyngeal space, especially, lymph nodes are frequently affected, although true retropharyngeal abscesses are apparently quite rare. According to our earlier observations, when the retropharyngeal space is affected in adults, it is most likely due to the spreading of infection from other deep spaces of the neck, usually from peritonsillar and parapharyngeal spaces. Children had significantly lower CRP than adults (Table 1), despite having similar rates of abscess formation. This highlights the significance of imaging and suggests that low CRP does not exclude an abscess in children. The specific risks related to MRI use include an excessive rise in patient body temperature (especially relevant for patients with high fever) and potential harm related to unknown magnetizing foreign body objects. These risks can be controlled with careful patient selection and preparation. Body temperature was not routinely measured during or immediately after MRI, but all the scans remained at the recommended specific energy absorption rate limits. The majority of the children received antipyretics before the MRI.

Sedation or general anesthesia is often required when MRI is performed on children to achieve sequences free of motion artifact. In our cohort, 16 (36%) patients were sedated (with spontaneous breathing) and only 3 (7%) patients required general anesthesia with intubation. All patients with general anesthesia had a serious course of illness including risk of airway compromise and a clinical need for monitoring vital functions. These patients were transferred either to the operating room or to ICU immediately after the MRI, without disruption of general anesthesia. According to patient records, no immediate complications related to sedation or general anesthesia occurred. Fifteen patients were sedated with thiopental and one with dexmedetomidine.

In the literature, a lively debate and expert opinions have been published for and against the use of CT and MRI in the pediatric population, and the argumentation is evolving [19]. In short, the scientific conversation focuses on weighing up the potential harm of radiation and the possible immediate or long-term harm related to anesthesia. Cost-effectiveness and limited MRI scanner availability are probably equally important variables in many institutions. MRI can be performed relatively quickly (20–30 min), but scan times are still longer than those of CT. Due to longer scanning times, even dedicated emergency MRI devices can be busy with other scans, which may cause further delay. Overall, these factors may prolong the length of stay in the emergency room and the time from presentation to diagnosis. The availability of MRI devices as well as that of pediatric anesthesiologists may be limited to larger institutions.

The limitations of this study include its retrospective nature and its relatively small number of pediatric patients. Even when the data collection period was more than five years, no more than 45 pediatric patients underwent MRI because deep neck infection was suspected. A further limitation of the current study is the lack of universally accepted criteria for true abscesses on MRI. For example, the interpretation of restricted diffusion is based on qualitative visual assessment. Despite these limitations, MRI diagnosis of an abscess has a high PPV and high interobserver agreement [16]. Data collection was comprehensive, and the number of pediatric patients was comparable to that of studies with similar objectives evaluating CT use.

## Conclusion

Emergency neck MRI is a feasible and accurate imaging method for diagnosing pediatric neck infections. According to our data and the previous literature, its diagnostic accuracy is superior to that provided by CT. Additional benefits of MRI, especially in children, include the lack of ionizing radiation. In younger age groups, retropharyngeal space infections and suppurative lymphadenitis are emphasized, reflecting the anatomical differences between children and adults. These differences require special attention and radiologic interpretation skills.

## Supplementary Information

Below is the link to the electronic supplementary material.

Supplementary file1 (DOCX 17.2 KB)

## References

[1] Vieira F, Allen SM, Stocks RM, Thompson JW (2008). Deep neck infection. Otolaryngol Clin North Am.

[2] Ogura I, Kaneda T, Kato M (2004). MR study of lateral retropharyngeal lymph nodes at different ages. Oral Surg Oral Med Oral Pathol Oral Radiol Endod.

[3] Chang L, Chi H, Chiu N-C (2010). Deep neck infections in different age groups of children. J Microbiol Immunol Infect.

[4] Maharaj S, Mungul S, Ahmed S (2020). Deep neck space infections: changing trends in pediatric versus adult patients. J Oral Maxillofac Surg.

[5] Broughton RA (1992). Nonsurgical management of deep neck infections in children. Pediatr Infect Dis J.

[6] Al-Sabah B, Bin Salleen H, Hagr A (2004). Retropharyngeal abscess in children: 10-year study. J Otolaryngol.

[7] Courtney MJ, Mahadevan M, Miteff A (2007). Management of paediatric retropharyngeal infections: non-surgical versus surgical. ANZ J Surg.

[8] Elden LM, Grundfast KM, Vezina G (2001). Accuracy and usefulness of radiographic assessment of cervical neck infections in children. J Otolaryngol.

[9] Vural C, Gungor A, Comerci S (2003). Accuracy of computerized tomography in deep neck infections in the pediatric population. Am J Otolaryngol.

[10] Rawlins KW, Allen DZ, Onwuka AJ, Elmaraghy CA (2019). Computed tomography use patterns for pediatric patients with peritonsillar abscess. Int J Pediatr Otorhinolaryngol.

[11] Malloy KM, Christenson T, Meyer JS (2008). Lack of association of CT findings and surgical drainage in pediatric neck abscesses. Int J Pediatr Otorhinolaryngol.

[12] Collins B, Stoner JA, Digoy GP (2014). Benefits of ultrasound vs. computed tomography in the diagnosis of pediatric lateral neck abscesses. Int J Pediatr Otorhinolaryngol.

[13] Choi SS, Vezina LG, Grundfast KM (1997). Relative incidence and alternative approaches for surgical drainage of different types of deep neck abscesses in children. Arch Otolaryngol Head Neck Surg.

[14] Côrte FC, Firmino-Machado J, Moura CP (2017). Acute pediatric neck infections: Outcomes in a seven-year series. Int J Pediatr Otorhinolaryngol.

[15] Freling N, Roele E, Schaefer-Prokop C, Fokkens W (2009). Prediction of deep neck abscesses by contrast-enhanced computerized tomography in 76 clinically suspect consecutive patients. Laryngoscope.

[16] Nurminen J, Velhonoja J, Heikkinen J (2021). Emergency neck MRI: feasibility and diagnostic accuracy in cases of neck infection. Acta Radiol.

[17] Muñoz A, Castillo M, Melchor MA, Gutierrez R (2001). Acute neck infections: prospective comparison between CT and MRI in 47 patients. J Comput Assist Tomogr.

[18] Virbalas J, Friedman NR (2021). Impact of neck CT on the management of suspected pediatric deep neck space infection. Int J Pediatr Otorhinolaryngol.

[19] Callahan MJ, Cravero JP (2021). Should I irradiate with computed tomography or sedate for magnetic resonance imaging?. Pediatr Radiol.

