# Supplementary material for: Magnetic resonance imaging findings in pediatric neck infections—a comparison with adult patients
Source: Pediatr Radiol. 2022 Feb 20;52(6):1158–66. doi: 10.1007/s00247-021-05275-6 (PMC9107440; doi:10.1007/s00247-021-05275-6)
Supplement: Supplementary file 1 — Supplementary file1 (DOCX 17.2 KB) [file 247_2021_5275_MOESM1_ESM.docx]

**Online Supplementary Material 1** Magnetic resonance imaging protocols used in the study.

| **Total *n*=371** | **Sequence** | **slice thickness, TE, TR, (b-value)** |
| --- | --- | --- |
| **Old protocol,**  ***n*=48 (13%)** | axial T2 TSE | 3 mm, 80 ms, 3,203 ms |
|  | coronal T2 SPAIR | 3 mm, 80 ms, 3,608 ms |
|  | sagittal T1 TSE | 3 mm, 16 ms, 641 ms |
|  | axial DWI | 4 mm, 86 ms, 4,843 ms, (1,000 s/mm^2^) |
|  | axial T1 SPIR post contrast (Gd) | 3 mm, TE=18 ms, TR=651 ms |
|  | coronal T1 TSE post contrast | 3 mm, TE=16 ms, TR=604 ms |
| **Novel protocol,**  ***n*=323 (87%)** | axial T1 TSE | 4 mm, 10 ms, 641 ms |
|  | axial T2 TSE Dixon | 4 mm, 100 ms, 3,021 ms |
|  | coronal T2 TSE Dixon | 3.5 mm, 80 ms, 3,210 ms |
|  | axial DWI | 4 mm, 87 ms, 3,981 ms (1,000 s/mm^2^) |
|  | axial T1 TSE Dixon post contrast (Gd) | 4 mm, 7 ms, 634 ms |
|  | coronal T1 TSE Dixon post contrast | 3.5 mm, 14 ms, 560 ms |
|  | sagittal T1 TSE Dixon post contrast | 3 mm, 14 ms, 630 ms |

*DWI* diffusion-weighted imaging, *Gd* gadolinium, *SPAIR* spectral adiabatic inversion recovery, *SPIR* spectral presaturation with inversion recovery, *TE* echo time, *TR* repetition time, *TSE* turbo spin echo
